# Phytochemical Analysis and *In Vitro* and *In Vivo* Pharmacological Evaluation of *Parthenium hysterophorus* Linn

**DOI:** 10.1155/2022/6088585

**Published:** 2022-06-17

**Authors:** Gul, Abdur Rauf, Imtiaz Ali Khan, Sulaiman Mohammad Alnasser, Syed Uzair Ali Shah, Md. Mominur Rahman

**Affiliations:** ^1^Department of Pharmacy, University of Swabi, Anbar 23561, Khyber Pakhtunkhwa, Pakistan; ^2^Department of Chemistry, University of Swabi, Anbar 23561, Khyber Pakhtunkhwa, Pakistan; ^3^Department of Entomology, The University of Agriculture, University of Peshawar, Peshawar, Khyber Pakhtunkhwa, Pakistan; ^4^Department of Pharmacology and Toxicology, Unaizah College of Pharmacy, Qassim University, Unayzah, Saudi Arabia; ^5^Department of Pharmacy, Faculty of Allied Health Sciences, Daffodil International University, Dhaka 1207, Bangladesh

## Abstract

The main aim of this research was to explore *Parthenium hysterophorus* Linn phytochemically and pharmacologically. Phytochemical screening is important for the isolation of active compounds before bulk extraction. The crude extracts and their fractions were screened for enzyme (urease, *α*-glycosidase, and phosphodiesterase) inhibition assays, *in vivo* analgesic, anti-inflammatory, and sedative effects. Results indicated the presence of steroids, flavonoids, etc. The crude extracts such as methanol, hexane, aqueous, ethyl acetate, chloroform, and butanol exhibited excellent urease inhibitory activities with IC_50_ = 43.1 ± 1.24, 31.9 ± 2.21, 31.9 ± 2.21, 57.3 ± 1.27, 49.2 ± 2.16, and 35.3 ± 1.12, respectively, as compared to standard acetohydroxamic acid (20.3 ± 0.43). The extracts (methanol, hexane, aqueous, ethyl acetate, chloroform, and butanol) also showed promising *α*-glycosidase potency with IC_50_ = 13.1 ± 0.34, 21.2 ± 1.16, 23.1 ± 0.12, 84.2 ± 2.17, 118.6 ± 3.07, and 840 ± 1.73, respectively against acarbose (840 ± 1.73). The phosphodiesterase activity of the mentioned extracts was also excellent with IC_50_ = 131.1 ± 2.41, 197.2 ± 3.16, 24.2 ± 0.11, 62.4 ± 2.21, 152.4 ± 1.81, and 55.3 ± 2.15, respectively, against the standard (265.5 ± 2.25). Furthermore, butanol (14.96 ± 1.78), ethyl acetate (18.98 ± 1.71), and methanol (16.87 ± 1.00) showed dose-dependent analgesic effects with a maximum inhibition of acetic acid-induced writhes. Whereas, methanolic and butanol extracts exhibited maximum inhibition of inflammation in the carrageenan paw edema test. The aqueous (*p* < 0.01) and butanol (*p* < 0.01) extracts exhibited maximum a sedative effect followed by chloroform (*p* < 0.05), ethyl acetate (*p* < 0.05), and methanolic (*p* < 0.05) fractions as compared to the standard drug. The current research concluded that *Parthenium hysterophorus* Linn has important phytochemical constituents having inhibitory effects on urease, *α*-glycosidase, and phosphodiesterase enzymes. Furthermore, the plant has analgesic, anti-inflammatory, and sedative effects. The *P. hysterophorus* needs to further be explored for the candidate molecules responsible for the abovementioned activities.

## 1. Introduction

*Parthenium hysterophorus L.* (*Asterales*, *Asteraceae*, Linn) is a flowering plant and an aggressive ubiquitous annual herbaceous weed belonging to the family *Asteraceae*. *P. hysterophorus* is commonly known as altamisa, bitter weed, white top, and carrot grass. The flowering of this plant occurs throughout the year [1, 2]. *P. hysterophorus* thrives in Pakistan, America, Africa, Asia, as well as in Australia. It has multi medicinal traditional usage such as for the treatment of fever, neurologic disorders, diarrhea, dysentery, malaria, urinary tract infections, allergic respiratory problems, mutagenicity in humans, and emmenagogue. In addition, *P. hysterophorus* has been employed in traditional medicine as a remedy for inflammation and rheumatism, and as an analgesic in muscular rheumatism [3]. This plant is rich in active compounds which are responsible for its use in traditional medicine. However, *P. hysterophorus* is not well-explored for its phytochemical constituents. Therefore, research is required for the isolation, purification, and structure determination of active constituents of this plant. *P. hysterophorus* has been reported to contain toxins called sesquiterpene lactones such as the glycoside parthenin [4]. Other phytotoxic compounds or allelochemicals present in this plant are hysterin, ambrosin, and flavonoids such as fumaric acid, quercetagetin 3,7-dimethylether, *p*-coumaric, *p*-hydroxybenzoin, vanillic acid, chlorogenic acid, anisic acid, ferulic acid, and various alcohols [5]. Ether and ethyl acetate fractions of *P. hysterophorus* have led to the isolation of fourteen compounds and some of them having cytotoxic potential [6]. A novel hydroxyproline-rich glycoprotein has been documented from the pollen of *P. hysterophorus* [7]. Another novel sesquiterpenoid, charminarone, has also been previously reported [8–10]. On the basis of the above-mentioned information, there is a need to fully explore the medicinal potential of *P. hysterophorus*, and to extract and isolate more active phytochemicals that rationalize its properties and pharmaceutical applications. The present study is an attempt to evaluate the *in vitro* enzyme (urease, *α*-glucosidase, and phosphodiesterase) inhibition assays, *in vivo* analgesic, anti-inflammatory, and sedative effects of the various fractions of *P. hysterophorus.*

## 2. Material and Methods

### 2.1. Plant Collection and Drying

Plant material of *P. hysterophorus* was collected from various areas of the University of Swabi, Anbar, K.P., Pakistan. The plant specimen was validated by a botanist in the Department of Botany, University of Swabi KP, Pakistan, and voucher specimen NO. BOT.UOS4 was deposited in the said department. The plant material was dried under shade at room temperature and on the ground to obtain a powder for extraction.

### 2.2. Extraction and Fractionation

The obtained *P. hysterophorus* powder material was subjected to cold extraction using a polar organic solvent such as methanol or ethanol, distilled water, and *n*-hexane. The extract was concentrated by means of a rotary evaporator at a low temperature (50–55°C). This was followed by fractionation using organic solvents of different polarities such as Hexane, CHCl_3_, EtOAc, and methanol (×3 for each). The extract was then suspended with the minimum amount of water, followed by the addition of different organic solvents starting from nonpolar (*n*-hexane) to more polar ones such as chloroform, dichloromethane, ethyl acetate, and butanol, respectively. Each collected fraction was concentrated under a vacuum by using a rotary evaporator at a low temperature (50–55°C) to obtain a crude fraction. The crude extracts and fractions were subjected to phytochemical screening before bulk extraction. Finally, the obtained crude extracts and fractions were subjected to *in vitro* and *in vivo* biological assays.

### 2.3. Phytochemical Screening

A phytochemical screening test of extracts/fractions was carried out for identification of active phytochemicals as per reported procedures [11–15].

### 2.4. *In Vitro* Enzyme Assays

#### 2.4.1. Urease Inhibition Activity

The urease inhibition activity of the crude extracts and fractions was performed by spectrophotometry in 96-well plates as per the standard method [16]. 5 *µ*L of crude extracts and their fractions (0.5 mM) and 25 *µ*L urease catalyst (1 U/well) were hatched for 15 minutes at 30°C. Then, 55 *µ*L substrate urea (100 mM) was re-brooded at 30°C for 15 minutes. After completion of incubation, 70 *µ*L of alkali reagents (0.1% sodium hypochlorite) and 45 *µ*L of phenol (0.005% w/v, sodium nitroprusside and 1% w/v phenol) were mixed. The incubation of the plates was again performed for 50 minutes at 30°C. The urease screening test was periodically done with continuous urea hydrolysis and ammonia production. The change in absorbance (optical density (OD)) was monitored at 630 nm on an ELISA plate reader (Spectra Max M2, Molecular Devices CA, (USA).

#### 2.4.2. *α*-Glucosidase Inhibitory Assay

The rat intestinal (CH_3_)_2_CO (acetone) powder in normal saline (100 : 1; w/v) was sonicated appropriately, and the supernatant was used as a source of basic intestinal *α*-glucosidase after centrifugation [17]. Shortly, 10 mL of the prepared extract and its isolated fractions of 5 mg/mL in DMSO solution was reconstituted in 100 mL of 100 mM phosphate buffer (pH 6.8) in 96-well microplates. The hatching was done in 50 mL of essential intestinal *α*-glucosidase for 5 min before 50 mL of substrate (5 mM p-nitrophenyl-a-D-glucopyranoside (p-NPG) was arranged in the similar buffer) was included. The *α*-glucosidase-mediated conversion of p-NPG into D-glucose and p-nitrophenol at 405 nm was monitored spectrophotometrically every 5 minutes. Singular seats for the screening extract were set up to extract baseline absorbance of the substrate and altered with 50 mL of buffer. The control sample contained 10 mL DMSO alongside screening samples. The percentage of enzyme inhibition was assessed as follows:

(1) $$\begin{array}{c} 1-\left(\frac{B}{A}\right)\times 100, \end{array}$$

where *A* speaks to the absorbance of the control exclusive of the prepared extract sample, and *B* connects to absorbance in the attendance of test samples.

#### 2.4.3. Phosphodiesterase Inhibitory Assay

This enzyme assay was performed using snake venom-derived PDE-1 (Sigma P-4631), adopting already published methods with some modifications [18]. 30 mM Mg-acetate and 33 mM Tris-HCl buffer (pH = 8.8) were mixed as a cofactor with 0.000742 U of enzyme in 96-well plates, then 0.33 mM bis (p-nitrophenyl) phosphate (Sigma N-3002) was added as a substrate. Ethylenediaminetetraacetic acid (EDT, E. Merck, Germany) was used as a standard drug. The inculcation was achieved for 30 minutes and the enzyme screening was examined at 37°C using a microtiter plate reader spectrophotometer, by the subsequent discharge of *p*-nitrophenol from *p*-nitrophenyl phosphate at 410 nm. All the screening tests were done in triplicate and the initial rates were calculated as the rate of changes in the OD/min (optical density/Min) and then used in the following calculation:

(2) $$\begin{array}{c} \%\text{ Inhibition}=100-\left(\frac{\text{OD testwell}}{\text{OD control}}\right)\times 100. \end{array}$$

### 2.5. *In Vivo* Biological Screening

#### 2.5.1. Analgesic Activity

BALB/c mice of both sexes (*n* = 6) weighing 18–22 g were used. All animals were withdrawn from food 3 hours before the start of the experiment and the mice were distributed in different groups. Among the divided animals, group I was injected with normal saline (10 ml/kg) as the control, while group II was administrated with the standard drug (diclofenac sodium; 10 mg/kg), and the rest of the groups were administered with various extracts and fractions (25, 50, and 100 mg/kg i.p). After administration of 30 min, the animals were treated with 1% acetic acid. Then, the number of abdominal constrictions (writhes) was counted after 5 min of acetic acid injection for the period of 10 minutes as per the usual methods [19].

#### 2.5.2. Anti-Inflammatory Activity

The crude extracts and various fractions of *P. hysterophorus* were also screened for anti-inflammatory activity as per the standard procedure [20]. The animals were divided into different groups of both sexes. Groups I and II were injected with normal saline (10 ml/kg) and diclofenac sodium (10 mg/kg) respectively, while the rest of the groups were administered with extracts/fractions at various doses (25, 50, and 100 mg/kg). After 30 minutes of intraperitoneal treatment, carrageenan (1%, 0.05 ml) was injected subcutaneously into the sub plantar tissue of the hind paw of each mouse. The inflammation was restrained using a plethysmometer (LE 7500 Plan Lab S.L) directly after injection of carrageenan, and then after 1, 2, 3, 4, and 5 hours of carrageenan injection. The regular foot swelling of drug-treated animals, as well as standard, was associated with that of control, and the percent inhibition of edema was calculated using the following formula:

(3) $$\begin{array}{c} \%\text{ Inhibition}=A-\left(\frac{B}{A}\right)\times 100, \end{array}$$

where *A* represents the edema volume of the control and *B* represents the paw edema volume of the tested group.

#### 2.5.3. Sedative Activity

The crude extracts and various fractions of *P. hysterophorus* were also screened for muscle relaxation activity. For this screening test, a 30 cm long Pyrex glass tube with a 3 cm diameter was used in this study. From the base, the design tube is marked at 20 cm and the animals were screened after 30, 60, and 90 minutes of treatment. Various groups (*n* = 5) were treated with normal saline (10 ml/kg), standard drug, and tested extracts and their fractions (5 and 10 mg/kg i.p). The animals were introduced at one edge of the tube and then permitted to move up to the mark 20 cm from the base. When the treated animals touched the 20 cm mark, the tube was moved straight to the perpendicular position and the animals strained to climb again to the tube with a backward effort. The mouse which failed to reach up to the mark within 30 seconds was considered to have relaxed muscles [20].

## 3. Results

Phytochemical analysis of *Parthenium hysterophorus* is given in Table 1. The crude extracts and fractions exhibited the presence of various secondary metabolites such as steroids, fatty acids, and terpenoids. These identified phytochemicals are responsible for its urease, *α*-glucosidase, and phosphodiesterase inhibitory effects.

### 3.1. *In Vitro* Enzyme Inhibition Assays

#### 3.1.1. Urease Inhibition

The urease inhibitory activities of this plant are given in Table 2. The crude extracts and various fractions of *Parthenium hysterophorus* showed excellent urease inhibition activity. The polar extracts such as ethyl acetate (87.3%), butanol (84%), and aqueous (81.4%) showed excellent activity with IC_50_ values of 57.3 ± 1.27, 35.3 ± 1.12, and 31.9 ± 2.21, respectively. The inhibitory potential is followed by *n*-hexane (77.4%), methanolic (74.2%), and chloroform (74.2%) fractions which showed moderate activity with IC_50_ values of 39.8 ± 0.36, 43.1 ± 1.24, and 49.2 ± 2.16, respectively. Acetohydroxamic acid (96.3%) is used as a standard urease inhibitor with an IC_50_ value of 20.3 ± 0.43.

#### 3.1.2. *α*-Glycosidase Inhibition

The *α*-glycosidase inhibitory activities of this plant are given in Table 3. The crude extracts and different fractions of *P. hysterophorus* showed excellent *α*-glycosidase inhibition potential. The extracts such as methanolic (94.2%), aqueous (91.4%), and butanol (90.2%) showed excellent activity with IC_50_ values of 13.1 ± 0.34, 23.1 ± 0.12, and 840 ± 1.73, respectively. The inhibitory potential of *α*-glucosidase was followed by *n*-hexane (84.7%), ethyl acetate (82.7%), and chloroform (76.4%) fractions with IC_50_ values of 840 ± 1.73, 84.2 ± 2.17, and 118.6 ± 3.07, respectively. The standard *α*-glucosidase inhibitor, acarbose, exhibited percent inhibition (90.2%) with an IC_50_ value of 840 ± 1.73.

#### 3.1.3. Phosphodiesterase Inhibition

The phosphodiesterase inhibitory activities of this plant are given in Table 4. The crude extract and different fractions of *P. hysterophorus* showed excellent phosphodiesterase inhibition potential. The extract such as aqueous (91.4%), butanol (88.4%) and n-hexane (82.7%) showed excellent activity with IC_50_ values of 24.2 ± 0.11, 55.3 ± 2.15, 197.2 ± 3.16, respectively. The inhibitory potential of phosphodiesterase is followed by the methanolic (79%), chloroform (78.4%), and ethyl acetate (76.5%) with IC_50_ values of 131.1 ± 2.41, 152.4 ± 1.81, and 62.4 ± 2.21, respectively. The standard phosphodiesterase inhibitor, EDTA exhibited 87.9% inhibition with an IC_50_ value of 265.5 ± 2.25.

### 3.2. *In Vivo* Activities

#### 3.2.1. Analgesic Effect

The crude extracts and various fractions of *P. hysterophorus* including methanol, hexane, aqueous, ethyl acetate, chloroform, and butanol were assessed for analgesic activity. All the extracts/fractions were tested at 25, 50, and 100 mg/kg and the effects were observed to be dose-dependent. Among the tested extracts, butanol, ethyl acetate, and methanol showed maximum analgesic effect as compared to the standard drug (Table 5). The aqueous, chloroform, and n-hexane fraction of *P. hysterophorus* exhibited a moderate analgesic effect (Table 5).

#### 3.2.2. Anti-Inflammatory Effect

**Figure 1:**
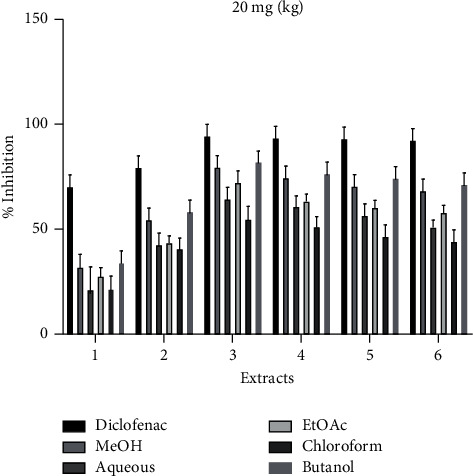
Anti-inflammatory activity of crude extracts/fractions of *P. hysterophorus*.

The crude extracts and various fractions were assessed for anti-inflammatory. Among the tested extracts, the methanolic and butanol extracts exhibited maximum inhibition after 3 hours of administration of extracts, as shown in Figure 1. However, the percent inhibition of the standard diclofenac sodium drug was promising throughout 6 hours of the experiment duration.

#### 3.2.3. Sedative Effect

The crude extracts and various fractions of *P. hysterophorus* including methanol, hexane, aqueous, ethyl acetate, chloroform, and butanol were assessed for sedative activity. Among the tested extracts, the aqueous and butanol extracts exhibited maximum sedative effect followed by chloroform, ethyl acetate, and methanolic fractions as compared to the standard drug (Table 6).

## 4. Discussion

Phytochemicals in medical plants play key roles in their biological potency. Phytochemical analysis plays a significant role in the isolation of new active and rare compounds. The biomedical importance of plants is correlated due to the presence of secondary metabolites. Our plant of interest, *P. hysterophorus,* was collected, processed, and extracted with various solvents to obtain crude extracts and fractions. The crude extracts and fractions exhibited the presence of various secondary metabolites such as steroids, fatty acids, terpenoids alkaloids, tannins, reducing sugars, saponins, flavonoids, and phlorotannins. *P. hysterophorus* is already reported as an ailment for various diseases in the traditional system. The plant is reported to have a diverse nature of compounds including allelochemicals (such as hysterin and ambrosin), flavonoids(such as fumaric acid, quercetagetin 3, 7-dimethylether, *p*-coumaric, *p*-hydroxybenzoin, vanillic acid, chlorogenic acid, anisic acid, and ferulic acid), and various alcohols [5]. A novel hydroxyproline-rich glycoprotein has been documented from the pollen of *P. hysterophorus* [7]. Another novel sesquiterpenoid, charminarone, has also been previously reported [8–10].

The extracts and fractions of *P. hysterophorus* were screened for in vitro selective enzyme inhibition assays including urease, *α*-glucosidase, and phosphodiesterase. The ethyl acetate (87.3%), butanol (84%), and aqueous (81.4%) fractions of *P. hysterophorus* exhibited maximum inhibition of urease enzyme compared to other fractions, however, exhibited less activity than the standard acetohydroxamic acid (96.3%). Urease is a metalloenzyme found in bacteria and plants. The urease inhibitors are important in the conditions associated with ureolytic bacteria [21]. Furthermore, the urease inhibitor is effective against *H pylori* infections [22]. In agriculture, urease inhibitors are associated with the proper utilization of urea fertilizers and modifying the nitrogen cycle [23]. Furthermore, *α*-glucosidase inhibition by various fractions of *P. hysterophorus* highlighted its importance for controlling hyperglycemia in diabetic patients [24, 25].

The phosphodiesterase inhibition by the fractions of *P. hysterophorus* is worth noting. Phosphodiesterase (PDE) has more than 40 isoforms, further subdivided into eleven families. These enzymes (present in each cell) hydrolyze the intercellular second messengers, cyclic nucleotide adenosine-3′, 5′- cyclic monophosphate (cAMP), and guanosine-3′, 5′-cyclic monophosphate (cGMP), thus altering cell response [24]. The PDEs are considered potential targets for combating various diseases including Alzheimer's disease, erectile dysfunction, and asthma [26]. The PDE isoforms expressed in cardiovascular systems and CNS are targeted in the treatment of pulmonary hypertension and cardiovascular disorders [27].

The acetic acid-induced writhing test is used to examine the preliminary analgesic properties of *P. hysterophorus* extracts and fractions [28]. The acetic acid causes the generation of pain mediators, thereby resulting in the constriction of the abdominal muscles [28, 29]. Our data exhibited promising analgesic effects in the butanol, ethyl acetate, and methanol extracts *P. hysterophorus*, whereas aqueous, chloroform, and *n*-hexane fractions exhibited moderate analgesic activity. Therefore, the phytochemical constituents of *P. hysterophorus* may play a role in inhibiting the release of pain-stimulating mediators such as prostaglandins (PGs), bradykinin, and histamine [30, 31]. Among these mediators, PGs are mostly responsible for the induction of pain. The selected plant probably inhibits cyclo-oxygenase, and thus, blocks the production of PGs and other intracellular cascades leading to pain, inflammation, and pyrexia.

The traction and chimney screening tests are common tools for the *in vivo* assessment of the skeleton muscle relaxation potential of substances [28]. In our findings, the aqueous and butanol extracts exhibited maximum sedative effect followed by chloroform, ethyl acetate, and methanolic fractions as compared to the standard drug, diazepam. The sedative and muscle relaxant effect suggested that the chemical constituents of this plant keep the anion neuronal channels open, especially the chloride channels which leads to central nervous system depression. The induction of anion influx is mostly related to the stimulation of GABA (gamma-aminobutyric acid) receptors, thereby hyperpolarizing the neuronal membrane via more chloride influx [32]. It is also suggested that the extracts or fractions might be accelerating the action of GABA neurotransmitters which are responsible for sedation and muscle relaxant effect.

## 5. Conclusion

It is concluded that crude extracts and various fractions showed the presence of active phytochemicals such as steroids, alkaloids, and terpenoids. The polar extracts and fractions exhibited excellent enzyme inhibition potency, thereby signifying the inhibition potential of urease, *α*-glucosidase, and phosphodiesterase by its phytochemical constituents. The sedative, anti-inflammatory, and analgesic potential of plants are also validated experimentally. Based on our data, we can conclude that *P. hysterophorus* could be a potential source of new, less toxic, safe, and more effective candidate drugs for pharmaceutical industries, thereby decreasing the economic burden for the therapy of different diseases involving the targets such as urease, *α*-glucosidase, and phosphodiesterase.

## Figures and Tables

**Table 1:** Phytochemical screening test of extracts and fractions of *P. hysterophorus.*

Phytochemicals	MeOH	Hexane	Aqueous	EtOAc	Chloroform	Butanol
Alkaloids	+	−	+	+	−	+
Tannins	+	−	+	+	−	+
Anthraquinones	−	−	−	−	−	−
Glycosides						
Reducing sugars	+	−	+	−	+	+
Saponins	+	−	+	−	−	+
Flavonoids	+	−	+	+	+	+
Phlobatannins	+	−	+	+	−	+
Steroids	+	+	+	+	+	+
Terpenoids	+	+	+	+	+	+

where MeOH is the methanolic extract and EtOAc is the ethyl acetate fraction.

**Table 2:** Urease inhibition activity of extracts and fractions of *Parthenium hysterophorus*.

Extract/fractions	Concentrations (*µ*g/mL)	% Inhibition	IC50 (*µ*g)
MeOH	0.2	74.2	43.1 ± 1.24
Hexane	0.2	77.4	39.8 ± 0.36
Aqueous	0.2	81.4	31.9 ± 2.21
Ethyl acetate	0.2	87.3	57.3 ± 1.27
Chloroform	0.2	74.2	49.2 ± 2.16
Butanol	0.2	84.0	35.3 ± 1.12
Acetohydroxamic acid	0.2	96.3	20.3 ± 0.43

**Table 3:** *α*-Glucosidase activity of extracts and fractions of *Parthenium hysterophorus.*

	Concentrations (*µ*g/mL)	% inhibition	IC50 (*µ*g)
MeOH	0.2	94.2	13.1 ± 0.34
Hexane	0.2	84.7	21.2 ± 1.16
Aqueous	0.2	91.4	23.1 ± 0.12
Ethyl acetate	0.2	82.7	84.2 ± 2.17
Chloroform	0.2	76.4	118.6 ± 3.07
Butanol	0.2	90.2	840 ± 1.73
Acarbose	0.2	90.2	840 ± 1.73

**Table 4:** Phosphodiesterase activity of extracts and fractions of *Parthenium hysterophorus.*

Extract/fractions	Concentrations (*μ*M) (*µ*g/mL)	% inhibition	IC50 (*µ*g)
MeOH	0.2	79.0	131.1 ± 2.41
Hexane	0.2	82.7	197.2 ± 3.16
Aqueous	0.2	91.4	24.2 ± 0.11
Ethyl acetate	0.2	76.5	62.4 ± 2.21
Chloroform	0.2	78.4	152.4 ± 1.81
Butanol	0.2	88.4	55.3 ± 2.15
EDTA	0.2	87.9	265.5 ± 2.25

where EDTA is for ethyl amine tetra acetic acid.

**Table 5:** The analgesic effect of crude extracts and various fractions of *P. hysterophorus*.

Treatment	Dose (mg/kg)	No. of writhing
Normal saline	10 ml/kg	65.77 ± 3.46
Diclofenac sodium	10	17.67 ± 1.14

MeOH	25	51.48 ± 2.45
	50	32.76 ± 1.19
	100	16.87 ± 1.00

Hexane	25	66.85 ± 2.41
	50	48.45 ± 1.17
	100	32.54 ± 1.99

Aqueous	25	60.87 ± 2.67
	50	41.45 ± 1.56
	100	25.45 ± 1.98

Ethyl acetate	25	55.54 ± 2.49
	50	33.76 ± 1.90
	100	18.98 ± 1.71

Chloroform	25	61.45 ± 2.40
	50	42.98 ± 1.14
	100	26.98 ± 1.98

Butanol	25	48.45 ± 2.41
	50	29.98 ± 1.98
	100	14.96 ± 1.78

**Table 6:** The sedative effect of crude extracts and various fractions of *P. hysterophorus*.

Treatment	Dose	No. of lines crossed
Normal saline	10 ml/kg	126 ± 1.25
Diazepam	0.5 mg/kg	6 ± 0.13^*∗∗∗*^

MeOH	25	110.51 ± 4.23
	50	102.60 ± 3.44
	100	94.68 ± 3.35^*∗*^

Hexane	25	115.90 ± 4.21
	50	108.69 ± 3.40
	100	100.99 ± 3.30^*∗*^

Aqueous	25	98.51 ± 4.29
	50	88.63 ± 3.24
	100	80.61 ± 3.14^*∗∗∗*^

Ethyl acetate	25	107.51 ± 3.99
	50	97.61 ± 3.29
	100	89.66 ± 3.00^*∗∗*^

Chloroform	25	105.55 ± 4.34
	50	94.65 ± 3.20
	100	83.69 ± 3.10^*∗∗*^

Butanol	25	100.54 ± 4.200
	50	90.69 ± 3.23
	100	81.65 ± 3.13^*∗∗∗*^

where, *P* < 0.05^*∗*^, *P* < 0.03^*∗∗*^, and *P* < 0.01^*∗∗∗*^.

## Data Availability

All data are available in the text.
